# Microbial-driven valorization of tannery sludge for fatty acids production: unraveling microbiome activity and functional redundancy

**DOI:** 10.3389/fmicb.2025.1675618

**Published:** 2025-10-06

**Authors:** Barbara Tonanzi, Alessio Massimi, Francesco Valentino, Giulia Adele Tuci, Marco Gottardo, Simona Rossetti, Simona Crognale

**Affiliations:** ^1^Water Research Institute, National Research Council of Italy, CNR-IRSA, Monterotondo, Rome, Italy; ^2^National Biodiversity Future Center, Palermo, Italy; ^3^Department for Innovation in Agroforestry and Biological Systems, University of Tuscia, Viterbo, Italy; ^4^Department of Environmental Sciences, Informatics and Statistics, Ca' Foscari University of Venice, Venice, Italy

**Keywords:** microbial community analysis, anaerobic sludge fermentation, short-chain fatty acids, tannery sludge, microbiome

## Abstract

The tannery sludge, with its high content of organic and inorganic compounds often hazardous to the environment, represents a major challenge in industrial waste management. Among the strategies to mitigate its impact, the biological production of fatty acids can be considered a cost-effective and sustainable solution. For the first time, this study investigated in depth the microbial community's composition and dynamics during anaerobic fermentation of tannery sludge to produce short-chain fatty acids (SCFAs). Systems with varying hydraulic retention times (HRT) and temperatures were explored, utilizing oxidative (Ox) and thermal (Th) pretreatments to enhance organic matter bioavailability. Mesophilic Ox reactors (Ox_4, Ox_8) achieved stable SCFAs production, primarily acetic acid. Ox_8, with longer HRT than Ox_4, showed higher organic matter conversion (0.32 ± 0.01 and 0.25 ± 0.01 g COD_SCFA_/g VS, respectively) and bacterial metabolic activity (FISH/CARD-FISH ratio of 0.8 and 0.9, respectively) as highlighted by microbiological analysis. Thermophilic Th reactors (Th_4, Th_8) yielded highest SCFAs production especially at longer HRTs (0.4 ± 0.01 g COD_SCFA_/g VS). The cutting-edge biomolecular approach herein applied has elucidated microbial community diversity and metabolic functionalities. Ox systems were dominated by *Actinobacteria, Bacteroidetes*, and *Firmicutes*, with *Proteiniphilum* as the key SCFAs producer. In contrast, Th reactors were mainly colonized by *Coprothermobacteraeota* and *Firmicutes*, with *Coprothermobacter sp*. playing a central role in SCFAs synthesis. Despite the compositional differences, both systems exhibited a noteworthy proteolytic functional redundancy, primarily linked to the substrate used. Among the first explorations, this research provides critical insights into the microbial adaptability and resilience of these biological systems.

## 1 Introduction

The leather tanning industry, a pivotal component of the global fashion and footwear sectors, is inseparably connected to extensive environmental challenges. The generation of substantial quantities of tannery sludge, a complex matrix of organic matter, toxic inorganic compounds, and microorganisms, poses a difficult threat to ecosystems due to its high organic load, heavy metal content, and recalcitrant nature (Abreu and Toffoli, 2009; Ali et al., 2015; Alibardi and Cossu, 2016). Due to the high content of organic and inorganic substances, often hazardous to the environment, this sludge poses a significant challenge for industrial waste management. Traditional waste management practices, such as landfilling and incineration, while seemingly expedient, intensify environmental issues by contributing to soil and water pollution, greenhouse gas emissions, and resource depletion (Alibardi and Cossu, 2016).

In particular, the Italian leather industry generates a significant amount of tannery sludge as a byproduct of leather processing. As outlined in the UNIC sustainability report of 2021, Italy's tannery sector is a global leader, accounting for 23% of the total global leather value (Unic Italian tannery 2021. UNIC Sustainability report). Italian legislation, particularly Legislative Decree 152/2006, strictly regulates the disposal of tannery sludge, requiring companies in the sector to adopt suitable technologies and treatment processes to minimize environmental impact. To reduce environmental impact and valorize these wastes, recent years have seen a growing interest in innovative solutions such as anaerobic digestion, co-composting, and biofuel production (Jaffari et al., 2024). These technologies allow for the transformation of tannery sludge into energy resources and useful bio-based materials, thus promoting a circular economy in the leather sector. In the pursuit of sustainable and circular economy principles, the valorization of tannery sludge emerges as a requiring imperative (Chojnacka et al., 2021; Moktadir et al., 2023). By transforming this waste into valuable products, the leather industry can mitigate its environmental impact while generating economic benefits. Microbial-based technologies represent a promising alternative for achieving this goal. Employing the metabolic capabilities of microorganisms, these technologies can effectively degrade complex organic matter, remove contaminants, and produce high-value products (Angenent et al., 2016; Bai et al., 2017; Ashraf et al., 2018; Saxena et al., 2020; Reyes-Romero et al., 2021; Ameen, 2023; Moktadir et al., 2023).

Fermentation and anaerobic digestion of tannery sludge align with the principles of circular economy. By transforming this waste into valuable bioproducts, these processes contribute to a more sustainable and resource-efficient leather industry. The anaerobic fermentation of tannery sludge produces short-chain fatty acids (SCFAs), which can be further converted into biofuels, bioplastics, or used as a nutrient source for algae cultivation (Han et al., 2019; Wu et al., 2019; Wang and Yin, 2022). This approach not only reroutes waste from landfills but also creates a circular pathway that recovers resources from a previously discarded material, thereby minimizing environmental impact and promoting economic growth. Pre-treatment of sludge prior to anaerobic process is a critical step to enhance microbial accessibility and overall process efficiency (Neyens et al., 2003; Appels et al., 2010; Braguglia et al., 2012; Gianico et al., 2013; Gallipoli et al., 2014; Gagliano et al., 2015; Khadaroo et al., 2019; Tonanzi et al., 2021). Physical, chemical, and biological pre-treatments can disrupt the complex organic matrix of sludge, increasing the surface area available for microbial attack and solubilizing recalcitrant organic compounds (Ariunbaatar et al., 2014; Chang et al., 2020; Gottardo et al., 2022; Wang and Yin, 2022). For instance, thermal hydrolysis has been shown to be effective in disrupting the sludge structure and increasing volatile solids solubilization (Gagliano et al., 2015; Gianico et al., 2015; Gherghel et al., 2019; Jeong et al., 2019). Furthermore, the combination of physical and chemical pre-treatments can synergistically enhance the performance of anaerobic processes (Neyens et al., 2003; Appels et al., 2010; Khadaroo et al., 2019; Li et al., 2023).

Recent improvements in biotechnological applications have shown the potential of microbial communities to address the complexities of tannery sludge (Jaffari et al., 2024). For instance, the application of anaerobic fermentation technology and oxidative or thermal pretreatment has shown high aptitude in minimizing landfilling by producing SCFAs, reducing organic matter content and solubilizing toxic compounds such as chromium, highly abundant in this waste (Zhai et al., 2020; Abate et al., 2021; Tuci et al., 2023, 2024; Zhu et al., 2024). These studies highlighted the potential of microbial-based approaches to transform tannery sludge from waste into a resource opportunity. Despite the key role of microorganisms in such process, the taxonomic diversity and functional potentialities of microbial communities involved in the production of fatty acids from tannery sludge have not been investigated so far. To effectively enhance biotreatment processes and product recovery from tannery sludge, a comprehensive characterization of the microbial community and its performance is necessary. For the first time, this study aims to characterize the taxonomic diversity and dynamics of the microbial communities involved in the anaerobic fermentation of tannery sludge in semi-continuous processes for the production of SCFAs. The insights gained will contribute to the development of innovative bioprocesses for a more sustainable and circular leather industry.

## 2 Materials and methods

### 2.1 Experimental set up

Microbiological analyses were performed on samples coming from SCFAs-producing semi-continuous reactors described in Tuci et al. (2023, 2024) (Table 1). These studies utilized a mixture of primary and secondary tannery sludge obtained from the Montebello Vicentino wastewater treatment plant (WWTP) in northeast Italy. Four different semi-continuous stirred tank reactors (sCSTRs) of 1.5 L working volume were operated to assess anaerobic fermentation of tannery sludge subjected to hydrogen peroxide (H_2_O_2_) or thermal pretreatment (Run Ox and Th, respectively). The reactors were inoculated with tannery sludge, maintained at 40°C and 50°C, respectively, and daily fed with pre-treated sludge for 1 week. A hydraulic retention time (HRT) of 4 and 8 days and organic loading rate (OLR) of 12.6 and 6.05 gVS/L d were tested in parallel (Table 1). Reactor performance was evaluated by monitoring SCFAs production, chemical oxygen demand (COD), ammonium, pH, and chromium concentration. The SCFAs composition, COD, volatile solids (VS) and other chemical parameters, including fermentation yield (Y), were analyzed according to the methods outlined in Tuci et al. (2023).

**Table 1:** Operational parameters of anaerobic reactors.

**Run**	**HRT (d)**	**OLR (gVS/L d)**	**Substrate pretreatment**	**Temperature (°C)**	**Reference**
Ox_4	4	12.6	0.4 g H_2_O_2_/g TS	40	Tuci et al., 2023
Ox_8	8	6.05	0.4 g H_2_O_2_/g TS	40	Tuci et al., 2023
Th_4	4	12.6	70°C, 20 h	50	Tuci et al., 2024
Th_8	8	6.05	70°C, 20 h	50	Tuci et al., 2024

HRT, hydraulic retention time; TS, total solids; VS, volatile solids.

### 2.2 *In situ* detection methods (FISH and CARD-FISH)

Samples were collected from the anaerobic systems and promptly fixed in a formaldehyde (2% v/v) and ethanol solution (48% v/v) before storage at −20°C. Prior to analysis, the fixed biomass was disaggregated by vortexing with glass beads. FISH and CARD-FISH analyses were performed using the EUB338mix oligonucleotide probe set (equimolar concentrations of EUB338, EUB338-II, and EUB338-III, http://www.probebase.net; Nielsen et al., 2009; Matturro et al., 2013; Tonanzi et al., 2018). Following hybridization, total cells were detected with DAPI using Vectashield Mounting Medium (Vector Labs, Italy). The abundance of fluorescent cells within the experimental samples was determined through the application of epifluorescence microscopy, a standard procedure consistently applied and documented in previous studies, including Tonanzi et al. (2018). The FISH/CARD-FISH ratio for each sample was determined as previously reported in Tonanzi et al. (2018).

### 2.3 Sample collection and processing for DNA extraction

Eight anaerobic samples were collected from each reactor throughout the experimental period. For DNA extraction, duplicate 2 mL aliquots were centrifuged as reported in Tonanzi et al. (2018), and the resulting pellets were stored at −20°C. The DNeasy PowerSoil Pro kit (QIAGEN, Hilden, Germany) was used for DNA extraction according to the manufacturer's instructions.

### 2.4 16S rRNA gene amplicon sequencing and data analysis

To characterize the microbial community structure, the 16S rRNA gene amplicon sequencing was performed using Illumina technology as previously described (Crognale et al., 2019). Briefly, the V1-V3 region of the bacterial 16S rRNA gene was amplified using primers 27F (5′-AGAGTTTGATCCTGGCTCAG-3′) and 534R (5′-ATTACCGCGGCTGCTGG-3′). Subsequent sequence data processing and analysis were conducted using QIIME2 (v. 2017.12), including demultiplexing, DADA2 quality filtering and trimming, and taxonomic assignment of amplicon sequence variants (ASVs) (Quast et al., 2013; Callahan et al., 2016). A total of 167,080 reads were obtained by high-throughput sequencing of the V1–V3 region of the bacterial 16S rRNA gene, that resolved into 4701 ASVs. The dataset is available through the Sequence Read Archive (SRA) under accession PRJNA1178871.

### 2.5 Statistical analysis

The statistical significance of the data was demonstrated through Pearson correlation coefficient, *t*-tests, and *p*-values (< 0.01). A non-metric multidimensional scaling (NMDS) ordination plot, based on the Bray-Curtis matrix, was performed to graphically synthesize the dissimilarity between samples. Process parameters and microbial community data were overlaid onto the NMDS plot using a vector-fitting procedure. Process data and values of major bacterial taxa revealed by 16S rRNA gene high-throughput sequencing (only genera ≥1% of total reads were considered) were normalized by log(X + 1). Statistical analyses were performed by using PAST software (Palaeontological STatistics, ver. 4.04) (Hammer et al., 2001).

## 3 Results and discussion

### 3.1 Impact of microbial activity on SCFAs production efficiency

**Figure 1:**
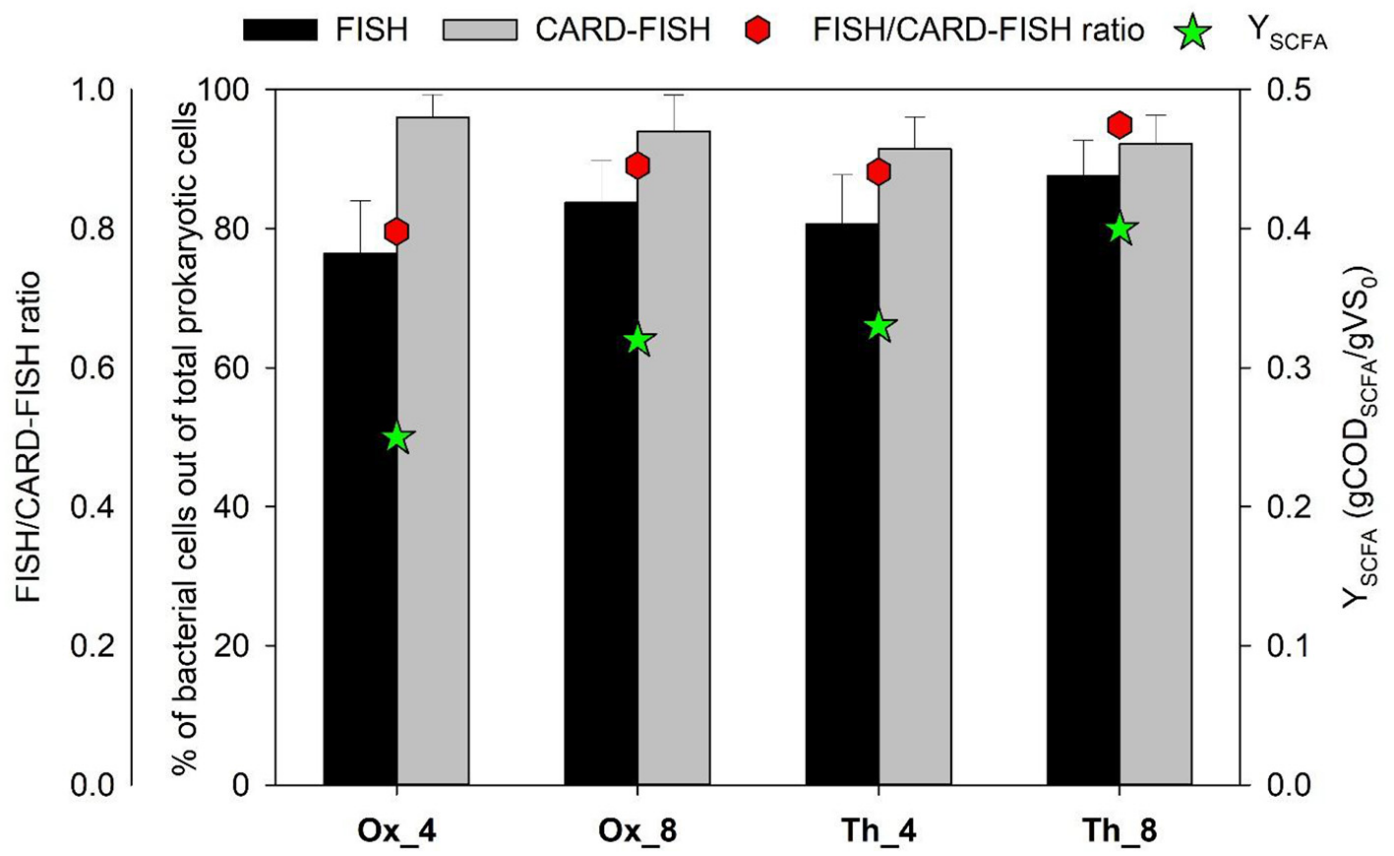
Bacterial abundance estimated by FISH and CARD-FISH analysis, FISH/CARD-FISH ratio and fermentation yield (Y). Values are expressed as: percentage of hybridized cells count out to total counts of DAPI-stained cells; ratio determined by dividing the average bacterial abundance estimated with FISH analysis by that obtained one with CARD-FISH analysis; ratio between the produced SCFAs over time and the initial VS concentration. Error bars indicate standard deviation.

As reported in the previous works of (Tuci et al. 2023, 2024), four semi-continuous reactors (Table 1) were operated at different HRT (4 and 8 days), sludge pretreatment (oxidative and thermal), OLR (6.05 and 12.6 gVS/Ld), and temperature (40 e 50°C). Fermentative performance and stability of the process were monitored by evaluating the production and profiles of SCFAs and the fermentation yield (Y), expressed as the ratio between the produced SCFAs over time and the initial VS concentration. In Ox systems, the Y obtained was higher in Ox_8 with respect to Ox_4 (0.32 ± 0.01 and 0.25 ± 0.01 g COD_SCFA_/g VS respectively) suggesting that a higher HRT associated with a lower OLR promoted a higher conversion rate of organic matter in SCFAs. This evidence was in line with the FISH/CARD-FISH ratio. As previously reported in Tonanzi et al. (2018), this ratio can be used as “gross parameter” to evaluate the active fraction of microorganisms in engineered biological processes. In fact, FISH analysis reveals physiologically active microorganisms in complex populations through the detection of cells with high ribosome content (>10^3^ ribosome per cell), while CARD-FISH analysis can be used to evaluate the total microbial community including the microbial components with low activity (few ribosomes per cell) (Pernthaler A. et al., 2002; Pernthaler J. et al., 2002). This parameter was here utilized to evaluate the fraction of active bacterial cells out of total members belonging to this domain. The FISH/CARD-FISH ratio estimated for bacteria over the operation is reported in Figure 1. In experiment Ox_4, a FISH/CARD-FISH ratio of 0.8 was observed, while in Ox_8, with a longer HRT, this ratio increased to 0.9 (Figure 1). These findings were statistically correlated with the estimated Y in these processes (*r* = 0.98, *p*-value < 0.01), supporting the observed similar trends.

A higher Y was observed in runs performed in Th systems, fed with thermally pretreated tannery sludge and operated under thermophilic conditions, compared to tests Ox_4 and Ox_8 (Figure 1). In particular, when a HRT of 8 days (run Th_8) was imposed, the Y value reached up to 0.4 ± 0.01 g COD_SCFA_/g VS. In this case as well, the detected FISH/CARD FISH ratio aligned with the trend of the Y, reaching a maximum of 0.95 in the Th_8 test (Figure 1). This high ratio highlighted a bacterial elevated metabolic activity, consistent with the higher yields achieved in this system.

Overall, the integrated evaluation of chemical and microbial metabolic data, in particular those obtained by *in situ* hybridization techniques, regarding the biological process of anaerobic fermentation of tannery sludge for SCFAs production was applied for the first time in this study. The findings indicated that longer HRTs were correlated with increased biomass activity, leading to improved conversion of organic matter to SCFAs. In addition, thermophilic conditions further enhanced bacterial activity, thereby increasing the resulting yield.

### 3.2 Bacterial dynamics in Ox and Th systems

**Figure 2:**
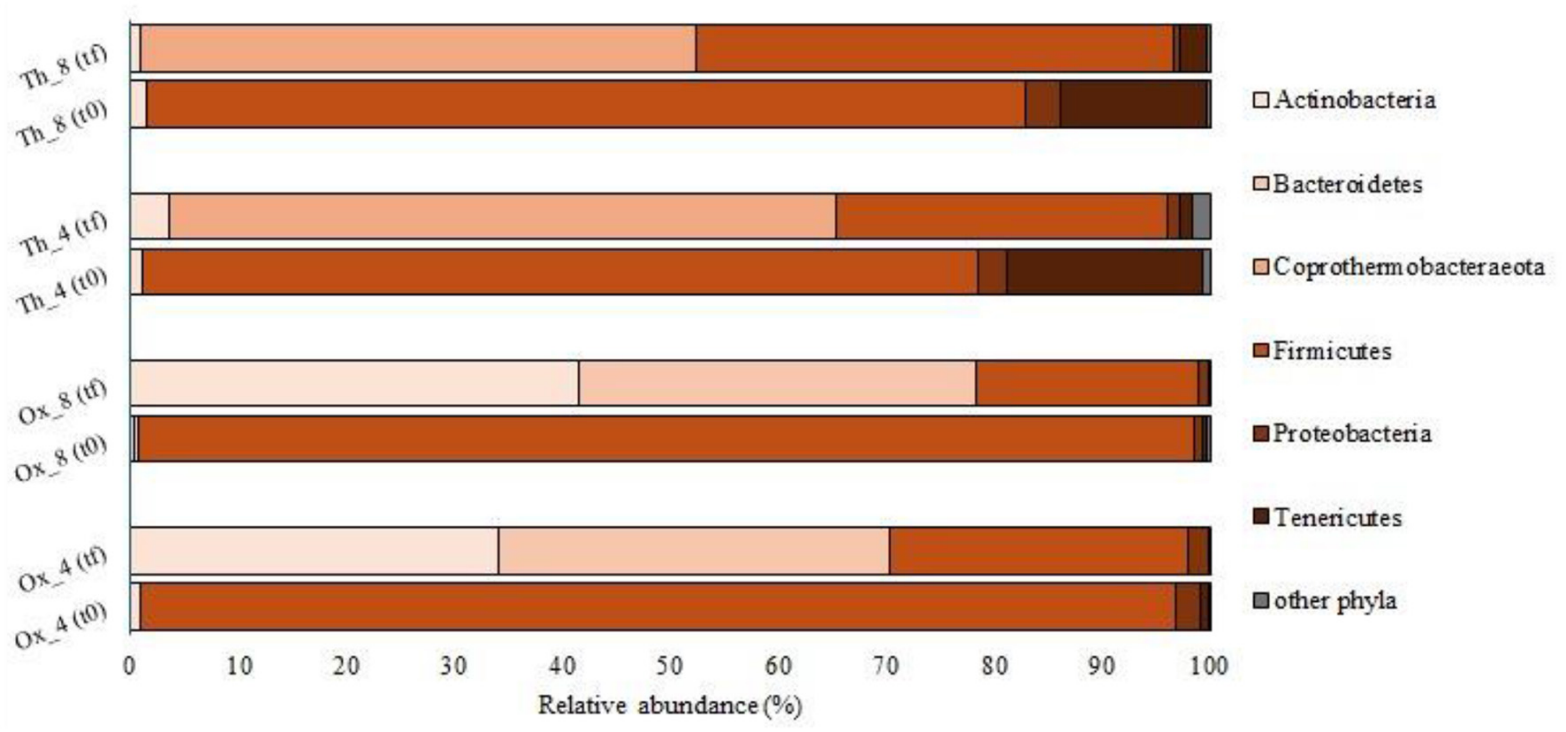
Bacterial community composition (% of total reads) at phylum level at the beginning (t0) and the end (tf) of the tests.

The bacterial taxonomic diversity was analyzed via 16S rRNA gene sequencing in samples collected from all experiments both at the beginning of the process and at steady state operation (t0 and tf, respectively). Tannery sludge was used as inoculum in all runs after 1 week of acclimatation. Sequencing analyses showed a massive microbial component attributable to the *Firmicutes* phylum (96–98% in Ox runs, and 77–81% in Th runs) at the initial time of all trials (t0, Figure 2). In particular, most of the sequences belonged to the *Clostridiales* order. The almost exclusive presence of these microorganisms can be explained mainly by the nature of the sludge used as inoculum, rich in organic matter and in particular proteins. Several studies have shown that members of the *Clostridiales* order were able to utilize protein and glucose to produce SCFAs (Zhang et al., 2019; Zhai et al., 2020). Furthermore, the phylum *Firmicutes* and in particular the class *Clostridia*, have been often linked to chromium contaminated environments (Branco et al., 2005; Yu et al., 2016). Indeed, the tannery sludge used in this study reported a total chromium (Cr) amount equal to 19.15 ± 0.3 g Cr/kg TS (Tuci et al., 2023, 2024), and this evidence may explain the high presence of this taxon at the beginning of the operations.

The bacterial composition observed in the runs using H_2_O_2_-pretreated sludge at both short (Ox_4) and long (Ox_8) HRTs during the steady state was similar. Almost all sequences belong to *Actinobacteria* (34% and 42%, for Ox_4 and Ox_8, respectively), *Bacteroidetes* (36% and 37%), and *Firmicutes* (28% and 21%), with a small portion (less than 2% in both experiments) attributable to the *Proteobacteria* phylum (Figure 2).

**Figure 3:**
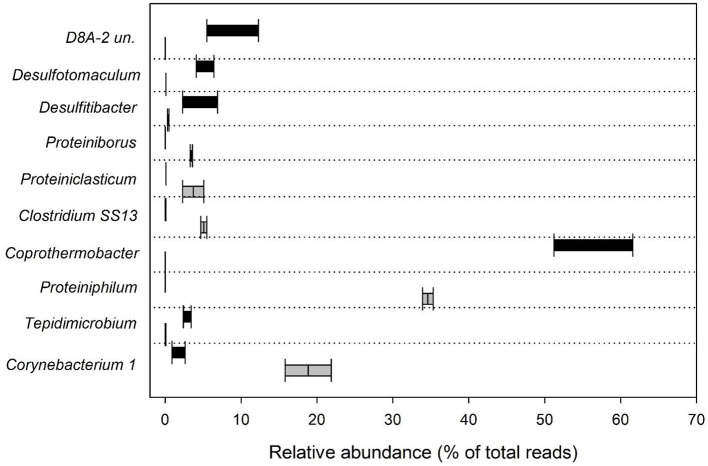
Box plot showing the relative abundance (% of total reads) of the 10 most abundant bacterial genera in Ox and Th runs.

Sequences affiliated with genus *Proteiniphilum* (*Bacteroidetes* phylum) represented 34 and 35% of the total reads in Ox_4 and Ox_8 tests, respectively (Figure 3). *Proteiniphilum sp*. is well known for its proteolytic metabolism aimed at producing SCFAs such as mainly acetate and propionate (Hahnke et al., 2016). The selective enrichment of this microorganism during the fermentation process was primarily influenced by the substrate composition. Its ability to efficiently degrade recalcitrant COD and rapidly adapt to nitrogen-rich organic matter, like proteins and collagen found in tannery sludge, facilitated its dominance within the microbial community (Zhu et al., 2024). In addition to the previously identified microorganisms, the reactors contained other microorganisms with similar metabolic functions (Dong et al., 2011; Yin et al., 2021). Notably, sequences belonging to genera *Proteiniborus, Proteiniclasticum* and *Clostridium sensu stricto 13* (*Firmicutes, Clostridia*) comprised between about 3 and 5% of the total reads obtained. The microbial community selected inside the Ox fermenters also highlighted the presence of genus *Corynebacterium 1* (*Actinobacteria*), which covered specifically 22% and 16% of the total reads in Ox_4 and Ox_8, respectively (Figure 3). Species belonging to *Corynebacterium* are chemoorganotrophs with a fermentative metabolism (Tauch and Sandbote, 2014) and have been extensively studied in tannery sludge in particular in relation to their resistance to Cr (Viti et al., 2003; Baba et al., 2020; Ameen, 2023).

Regarding the experiments conducted under thermophilic conditions with the feed sludge pretreated at high temperature (Th_4 and Th_8), most of the sequences belonged to the phylum *Coprothermobacteraeota* (62% and 51%, for Th_4 and Th_8, respectively), followed by the phylum *Firmicutes* (31% and 44%). In these experiments, sequences attributable to *Proteobacteria* (less than 1%) and to *Tenericutes* (1% and 3%) were also found (Figure 2). Most of the reads were affiliated with genus *Coprothermobacter sp*. (61.6% and 51% in Th_4 and 8, respectively). The latter, isolated for the first time from tannery sludge, is a fermentative proteolytic bacterium (Kersters et al., 1994; Gagliano et al., 2015; Pavan et al., 2019). Furthermore, the genus *D8A-2* (*Firmicutes, Clostridia*) was found to comprise between 5.4% and 12.3% of the total sequences in Th_4 and Th_8, respectively (Figure 3). This taxon has been previously reported in thermophilic anaerobic systems characterized by high levels of SCFAs, where it is thought to play a role in syntrophic interactions, especially in the presence of elevated ammonia concentrations (Lee et al., 2019; Li et al., 2022). The prominent presence of *Clostridia D8A-2* in these systems can once again be attributed to its ability to metabolize proteins. Indeed, the HRT in Th_8 facilitates the degradation of proteins, leading to a higher production of ammonia compared to Th_4 (Tuci et al., 2024), which creates a more favorable environment for the growth of this microorganism. In addition, the Th reactors contained other fermentative bacteria, such as *Desulfitibacter* (2.3% and 6.9%, respectively) and *Desulfotomaculum* (4.1 and 6.4% in Th_4 and 8). Certainly, *Desulfotomaculum* genus is of particular interest since it is known to employ metals such as Cr as terminal electron acceptors, and is capable of oxidizing H_2_ while reducing CO_2_ to acetate (Tebo and Obraztsova, 1998). As reported in previous studies, *Desulfitibacter* and *Desulfotomaculum spp*. are associated with the oxidation of sulfur in anaerobic digestion processes (Tebo and Obraztsova, 1998; Nielsen et al., 2006). As a consequence, the presence of this type of metabolic activity in the herein studied systems could also reduce the toxic effects of sulfides and heavy metal (e.g., Cr) on other microorganisms, as previously demonstrated by Zhu et al. (2024). Moreover, *Tepidimicrobium* genus was retrieved in both Th runs (2.4% and 3.4% of the total reads in Th_4 and 8, respectively). The presence of this microorganism in processes involving the use of tannery sludge is well documented, with studies specifically focusing on microbial analysis in soils contaminated by tannery sludge (Kong et al., 2019). This taxon had a strong adaptability and is usually observed in high organic-containing sewage sludges where it uses both carbohydrates and peptide compounds to produce acetate, ethanol, butyrate, hydrogen and carbon dioxide (Niu et al., 2009; Kong et al., 2019).

**Figure 4:**
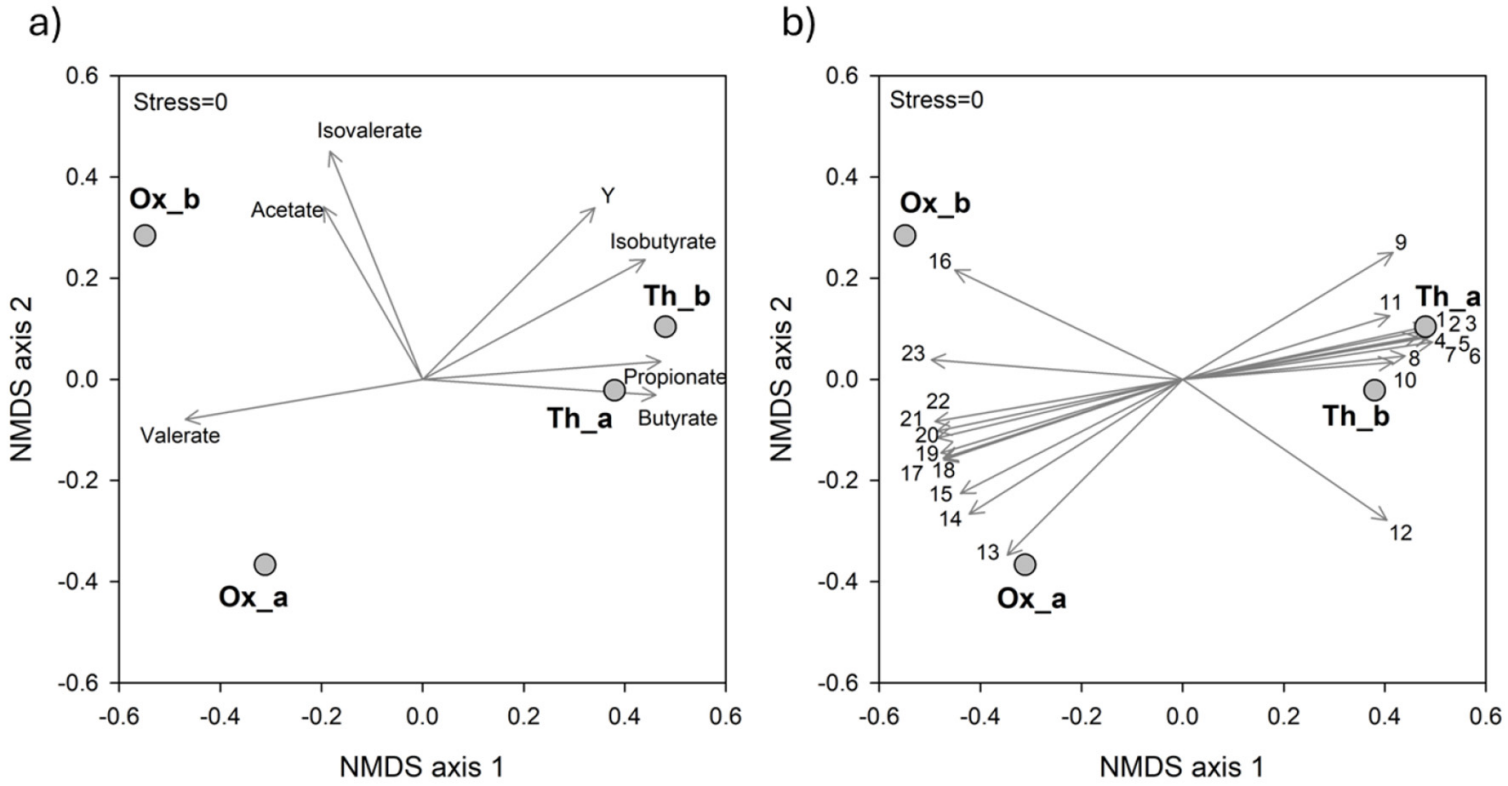
NMDS ordination plots, based on Bray-Curtis distance matrixes of log(X+1) transformed data. The vector length is proportional to the correlation between the NMDS axes and each parameter. The stress value (i.e., <0.2) suggests an accurate representation of the dissimilarity among samples. **(a)** The variation patterns of process parameters during the operation. **(b)** The relative abundance of the main genera (≥1% in at least one sample) is projected onto the NMDS ordination synthesizing the microbiological dissimilarity. 1, *Tepidimicrobium*; 2, *Desulfotomaculum*; 3, *Ruminococcaceae* unidentified; 4, *Izimaplasmatales* unidentified; 5, *Coprothermobacter*; 6, *Clostridia D8A-2* unidentified; 7, *Desulfitibacter*; 8, *Caldicoprobacter*; 9, *Firmicutes* unidentified; 10, *Clostridium sensu stricto 5*; 11, *Haloplasma*; 12, *Lutispora*; 13, *Dysgonomonas*; 14, *Clostridium sensu stricto 7*; 15, *Proteiniclasticum*; 16, *Bacillaceae* unidentified; 17, *Peptostreptococcaceae* unidentified; 18, *Corynebacterium 1*; 19, *Brevibacterium*; 20, *Clostridium sensu stricto 13*; 21, *Proteiniborus*; 22, *Proteiniphilum*; 23, *Coriobacteriales unidentified*.

Overall, the experimental conditions imposed in this study allowed a clear differentiation between Ox and Th systems both in terms of SCFAs production and bacterial selection, as outlined by NMDS analysis (Figure 4). In particular, Ox systems were characterized by a highest production of acetate and valerate, while Th systems by a highest production of butyrate and propionate and an overall highest Y. This clusterization was reflected also on microbiological dissimilarity among the two systems. In particular, the genera *Brevibacterium, Clostridium sensu stricto* 13, *Proteiniborus, Proteiniphilum* and *Coriobacteriales* unknown (vectors 19, 20, 21, 22, and 23, respectively) were significantly related to Ox runs. Conversely, *Tepidimicrobium, Desulfotomaculum, Ruminococcaceae* unknown, *Iziplasmatales Coprothermobacter* and *Clostridia D8A-2* (vectors 1, 2, 3, 4, 5, and 6, respectively) were significantly correlated with Th systems (Figure 4).

The effect of HRT on SCFAs production has been well documented in literature (Scoma et al., 2012; Jankowska et al., 2018; Aboudi et al., 2023; Chen et al., 2024; Gonçalves et al., 2024; Jimenez-Paez et al., 2025), generally showing that longer HRTs allow microorganisms more time to break down complex substrates, leading to higher SCFAs yields. In line with these evidences, this work highlighted that longer HRT enhanced microbial activity and fermentation yield during tannery sludge hydrolysis, while it did not significantly alter the community composition.

The distinct microbial communities selected in Ox and Th fermenters mainly resulted from the different incubation temperatures acting as the primary stressor. Indeed, temperature can significantly influence microbial growth and activity, affecting enzyme kinetics, membrane fluidity, and cellular stability (Knapp and Huang, 2022). As expected, higher temperature promoted thermophilic or thermotolerant microorganisms, while lower temperature favored mesophilic organisms. These temperature-driven differences can have implications for fermentation, affecting product yields and substrate conversion rates. Nevertheless, while microbial taxa diverged among the different tested systems mainly due to the temperature, the overall functional outcome remained consistent. Interestingly, although different microbial communities were selected under Ox and Th conditions, both systems exhibited the occurrence of various proteolytic microorganisms most likely responsible for the observed SCFAs production, highlighting the occurrence of functional redundancy and suggesting that multiple taxa can fulfill similar metabolic roles.

## 4 Conclusions

This study marks a significant advance by providing the first in-depth exploration of the microbial community dynamics and metabolic activity responsible for SCFAs production from tannery sludge. Our findings demonstrate that both oxidative and thermal pretreatments of tannery sludge led to high SCFAs production, particularly in systems with a low OLR (6.05 gVS/Ld) and a long HRT (8 d), correlated with the enhancement of microbial metabolic functionalities. Furthermore, process temperature emerged as a critical factor, profoundly influencing the selection of specific microorganisms essential for organic matter conversion to fatty acids. This parameter, coupled with the protein-rich substrate used, strongly drove the microbial community dynamics. In response to the operating conditions tested, a distinct core microbiome was selected among Ox and Th systems, consistently dominated by proteolytic microorganisms such as *Proteiniphilum, Proteiniclasticum*, and *Proteiniborus*. The observed presence of taxonomically diverse bacteria exhibiting similar metabolic capabilities strongly suggests a powerful implication: highly performing SCFAs-producing microbial consortia can be strategically selected and enriched to optimize fatty acid production. By exploiting the specific metabolic capabilities of these specialized microorganisms, it is possible to significantly improve the efficiency and yield of SCFAs synthesis processes. This microbiological understanding provides a foundational basis for designing and operating more effective bioreactors for waste valorization.

## Data Availability

The datasets presented in this study are publicly available. This data can be found here: https://www.ncbi.nlm.nih.gov, accession Number PRJNA1178871.
